# Physician Attitudes Toward Homosexuality and HIV: The PATHH-III Survey

**DOI:** 10.1089/lgbt.2018.0041

**Published:** 2018-10-15

**Authors:** Robert Marlin, Ankita Kadakia, Brandon Ethridge, William C. Mathews

**Affiliations:** ^1^ Department of Medicine, University of California, San Diego, San Diego, California.; ^2^ San Diego County Medical Society, San Diego, California.

**Keywords:** HIV, homosexuality, LGBT, physician attitudes, stigma, transgender

## Abstract

***Purpose:*** The aims of this study were (1) to evaluate current physician attitudes toward homosexuality and homosexual, transgender, and HIV-positive individuals and (2) to compare current attitudes of those from prior surveys of the same population, the San Diego County medical community.

***Methods:*** An online survey was conducted during November–December 2017 to assess general attitudes toward homosexuality and medically focused items that addressed homosexual orientation, transgender identity, and HIV. Responses were weighted for nonresponse. Predictors of stigma were assessed using generalized linear models. Trends across three surveys of the same population in 1982, 1999, and 2017 using common items were assessed using unweighted responses.

***Results:*** Of 4418 eligible physicians, 491 (11.1%) responded (median age 55 years, 38% female and 8.7% gay or bisexual). Regarding admission to medical school, 1% opposed admitting a homosexual applicant, 2% a transgender applicant, and 5% an HIV-positive applicant. Regarding consultative referral to a pediatrician, 3% would discontinue referral to a homosexual pediatrician, 5% to a transgender pediatrician, and 10% to an HIV-positive pediatrician. Regarding discomfort treating patients, 7% reported discomfort treating homosexual patients, 22% transgender patients, and 13% HIV-positive patients. Earlier year of graduation from medical school, male gender, and heterosexual orientation were significant predictors of stigma-associated responses. Compared with the results from surveys in 1982 and 1999, the current results suggest substantively less stigma associated with homosexuality and HIV.

***Conclusion:*** There have been substantive declines over a 35-year period in the prevalence of stigmatizing attitudes toward sexual minorities and HIV-positive people among physician respondents in three survey waves of the San Diego County medical community.

## Introduction

Recent research has documented important health disparities experienced by LGBT persons in the United States across several health conditions, health behaviors, healthcare access, and utilization characteristics.^1,2^ Stigma has emerged prominently as a potent contributor to at least some of the documented health disparities and may causally relate to disparities both through enactments of stigma by healthcare workers or through consequences of internalized homonegativity.^3–6^ Stigma can have detrimental effects on both healthcare access and quality.^7,8^ Although self-reported attitudes of healthcare providers do not necessarily predict professional or clinical behavior, they are measurable indicators that can inform evaluations of cultural competence to treat patients and to interact with colleagues who are at risk for prejudice.

A recent comparative effectiveness review concluded that the term cultural competence is not well defined for LGBT populations and that most cultural competence intervention studies did not “measure the downstream effect of changing provider beliefs on the care delivered to patients.”^9^ Nonetheless, there is theoretical support in Ajzen's theory of planned behavior for the premise that clinician attitudes relate causally to subsequent clinical behavior through the mediation of behavioral intention.^10,11^ Furthermore, there is empirical evidence that stigma-associated attitudes of physicians and health professional students predict behavioral intention to discriminate.^12,13^

Surveys of the membership of the San Diego County Medical Society (SDCMS) regarding attitudes toward homosexuality were conducted in 1982 and 1999, a period that covered the early and established phases of the HIV epidemic in the United States.^14,15^ We conducted a third survey wave of the SDCMS and University of California, San Diego (UCSD), clinical faculty in 2017 to assess further trends in attitudes toward homosexuality and homosexual, transgender, and HIV-positive individuals.

## Methods

### Sample

A 25-item anonymous Internet survey of physician members of the SDCMS and clinical faculty of the UCSD School of Medicine was conducted using the SurveyGizmo platform between November and December 2017 to assess attitudes toward homosexuality, transgender issues, and HIV. The sampling frame included all SDCMS (*n* = 3337) and UCSD (*n* = 1081) physicians who had antecedently given permission to be included in an e-mail registry maintained by the SDCMS, excluding 621 (14.7%) members with no e-mail on file plus an additional 288 (6.8%) who declined e-mail contacts from the SDCMS. A cover letter of invitation to participate signed by the SDCMS President, including a clickable link to the online survey instrument, was e-mailed to all physicians in the sampling frame on November 13, 2017, and was followed by two reminder invitations on November 30 and December 15, 2017.

### Measures

The survey instrument (Appendix 1) ascertained the following demographic characteristics: medical specialty, gender, gender identity, sexual orientation, year of medical school graduation, current practice setting, and SDCMS membership status. The survey included an 8-item 5-category Likert-type attitudinal scale (Heterosexual Attitudes Toward Homosexuality [HATH]-8), composed of 7 items of previously demonstrated high intrinsic consistency reliability from Larsen's 20-item HATH scale plus an additional item concerning attitude toward same-sex marriage.^15,16^ HATH-8 item responses were averaged with a possible scale range from 1 (most homo-favorable) to 5 (most homo-unfavorable).

The survey then explored medically oriented attitudes of respondents: (1) attitudes toward admission to medical school of homosexual, transgender, and HIV-positive applicants (1 = no, 0 = yes) and (2) behavioral intention to discontinue (1 = discontinue) or continue (0 = continue) consultative referral upon learning that a consultant physician in each of four specialties (pediatrics, general surgery, psychiatry, and radiation therapy) was homosexual, transgender, or HIV positive. Finally, the survey ascertained affective orientation (0 = no negative feelings, 1 = sometimes uncomfortable, 2 = often uncomfortable) in treating homosexual, transgender, or HIV-positive patients.

For each target group (homosexual, transgender, and HIV positive), medically oriented attitudes were combined as medical homosexual, transgender, and HIV-stigma indices, respectively, by summing responses across the six medically oriented items (medical school admission [one item], consultative referral [four items], affective orientation toward patients [one item; re-coded: 0 = no negative feelings, 1 = sometimes/often uncomfortable]) for each target group, with possible observable ranges of 0 (most favorable) to 6 (most unfavorable). Scales scores (HATH-8, Medical Homosexual Stigma [MHS], Medical Transgender Stigma, and HIV Stigma [HIVS]) were formed using two metrics: (1) the mean of summed included items and (2) percent of maximum possible (POMP).^17^

Consistent with recent recommendations, we have abandoned the previous terminology incorporating the term “phobia” with reference to unfavorable attitudes and have adopted the term “stigma,” itself further categorized as endorsed stigma (on survey items), internalized self-stigma, and expressed (acted out) stigma.^18^ So as to maintain consistency among measures common to all three survey waves, the ambiguous terms “homosexuals” and “homosexuality” were included in the 2017 survey, recognizing that these terms are no longer preferred and lack specificity regarding orientation, identity, and behavior. Likewise, the use of the term “transgender” without further descriptors in the 2017 survey does not capture the complexity of the concept nor of the lived experience.

Finally, because of the availability of data sets from the two preceding SDCMS surveys regarding physician attitudes toward homosexuality,^14,15^ we present a comparison of respondent attitudes using a metric of seven items from the original HATH scale that were used in all three survey waves, HATH-7. For ease of interpretation, HATH-7 scores are presented as POMP scores. In addition, we compare, across the three survey waves, responses to selected common individual items dealing with medical school entry and consultative referral. Because nonrespondent weights were available only for the current (2017) survey wave, the HATH-7 comparisons are based on unweighted scores for all three survey waves (1982, 1999, and 2017). The respondent numbers (response rates) for the 1982 and 1999 survey waves were 1009 (42.7%) and 736 (13%), respectively.^14,15^

### Statistical analyses

Statistical analyses were performed using the survey (svy) suite of functions in Stata 15.1 (StataCorp LLC, College Station, TX). The SDCMS provided selected demographic characteristics (gender, year of medical school graduation, practice setting, medical specialty, and SDCMS membership status) for both respondents and nonrespondents to the survey. These five characteristics were incorporated as inverse probability of response weights, estimated by a logit model of response, to adjust estimates for nonresponse bias. Estimates were further rake calibrated to the marginal totals of the sampling frame. Standard errors were estimated using the jackknife procedure.^19^

Scale intrinsic consistency reliability was estimated as Cronbach's alpha. Spearman's rho was used to estimate correlation among composite measures. Bivariate associations between attitudinal outcome measures and available demographic predictors were analyzed using contingency tables for categorical and ordinal comparisons and analysis of variance for continuous outcomes. Statistically significant results were defined using *p* < 0.05 as criterion. Because of positive skewness of the composite outcome measures, generalized linear models (GLM) using the gamma distribution with log link, adjusted for survey design characteristics, were fit to evaluate independent effects of characteristics found to be associated with outcomes in bivariate analyses.^20^

Association between selected binary-coded responses dealing with medical school entry, affective orientation about treating patients, and consultant referral, by referent characteristics (student, physician, or patient), was examined using the McNemar matched pair analysis.

## Results

Of 4418 physicians in the sampling frame, 513 (11.6%) clicked on the survey link embedded in the letter of invitation to participate. Of the 513, 491 (11.1% of 4418) submitted either complete (*n* = 460) or partial (*n* = 31) responses to all items on the survey.

### Comparison of participants with nonparticipants

Table 1 compares the characteristics of those who either clicked (participants) or did not click (nonparticipants) on the invitation letter survey link. Participants were older, graduated from medical school earlier, were more likely to be members of the SDCMS, and were more commonly female and Caucasian. Self-reported gender identity and sexual orientation were available only for survey respondents. One respondent (0.2%) identified as transgender (2 declining to state) and 41 (8.7%) identified as homosexual or bisexual (8 declining to state).

**Table 1.** Characteristics of Survey Participants and Nonparticipants

*Characteristic*	*Nonparticipant*	*Participant*	*Total*	p
*n* (%)	3905 (88.4)	513 (11.6)	4418 (100.0)	
Age, median (interquartile interval)	50 (40; 61)	55 (44; 65)	50 (40; 62)	<0.01
Medical school graduation year, median (interquartile interval)	1996 (1983; 2007)	1990 (1979; 2004)	1995 (1983; 2007)	<0.01
SDCMS member, *n* (%)				0.02
No	987 (25.3)	105 (20.5)	1092 (24.7)	
Yes	2918 (74.7)	408 (79.5)	3326 (75.3)	
UCSD affiliation, *n* (%)				0.30
No	2959 (75.8)	378 (73.7)	3337 (75.5)	
Yes	946 (24.2)	135 (26.3)	1081 (24.5)	
Gender, *n* (%)				0.04
Male	2472 (63.3)	307 (59.8)	2779 (62.9)	
Female	1287 (33.0)	194 (37.8)	1481 (33.5)	
Undisclosed	146 (3.7)	12 (2.3)	158 (3.6)	
Race/ethnicity, *n* (%)				<0.01
Caucasian	1235 (31.6)	210 (40.9)	1445 (32.7)	
Latino/Hispanic	129 (3.3)	21 (4.1)	150 (3.4)	
Black	23 (0.6)	1 (0.2)	24 (0.5)	
Asian/Native American/Pacific Islander	305 (7.8)	34 (6.6)	339 (7.7)	
Other	21 (0.5)	2 (0.4)	23 (0.5)	
Undisclosed	2192 (56.1)	245 (47.8)	2437 (55.2)	
Specialty groups, *n* (%)				0.04
Anesthesiology	296 (7.6)	37 (7.2)	333 (7.5)	
Dermatology	82 (2.1)	8 (1.6)	90 (2.0)	
Emergency Medicine	136 (3.5)	22 (4.3)	158 (3.6)	
Family Medicine/General Practice	287 (7.3)	37 (7.2)	324 (7.3)	
Internal Medicine	997 (25.5)	122 (23.8)	1119 (25.3)	
Neurology	100 (2.6)	11 (2.1)	111 (2.5)	
Obstetrics and Gynecology	171 (4.4)	28 (5.5)	199 (4.5)	
Pathology	69 (1.8)	11 (2.1)	80 (1.8)	
Pediatrics	259 (6.6)	50 (9.7)	309 (7.0)	
Psychiatry	163 (4.2)	29 (5.7)	192 (4.3)	
Radiology	203 (5.2)	28 (5.5)	231 (5.2)	
Surgical specialties	606 (15.5)	71 (13.8)	677 (15.3)	
Other	114 (2.9)	23 (4.5)	137 (3.1)	
Undisclosed	422 (10.8)	36 (7.0)	458 (10.4)	

SCDMS, San Diego County Medical Society; UCSD, University of California, San Diego.

### Survey measures

**FIG. 1.**
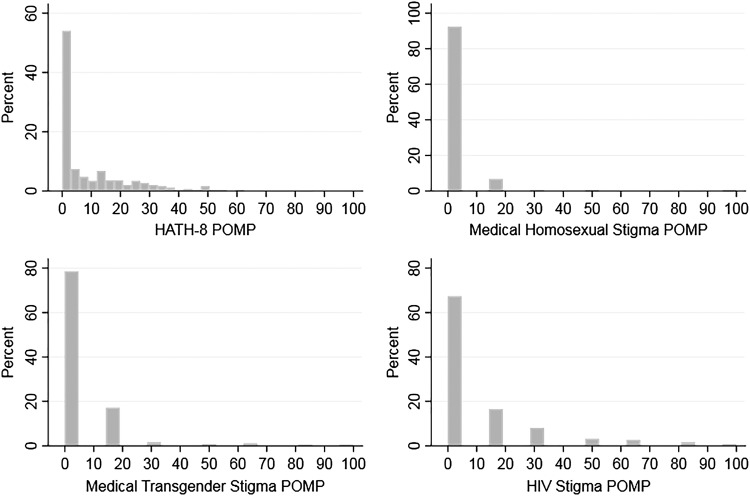
Distribution of composite outcome measures.

Table 2 presents the univariate item and scale distributions. For items with binary response options (0 = favorable; 1 = unfavorable), the reported column means may be interpreted as the proportions of respondents expressing unfavorable attitudes (Table 2). Scale scores are reported both as the mean of summed items and as POMP. The distribution of composite outcome score was highly skewed to the right (positively skewed), with most respondents scoring toward more favorable score ranges (Fig. 1). There was moderate correlation among the composite study measures (Table 2, footnote c).

**Table 2.** Univariate Distribution of Weighted Survey Items and Summary Scales

*Item or scale*	*Mean*	*SE*	*Observed range*	N	*Possible values*^a^
HATH-8 items					
Homosexuality is normal	1.72	0.06	1–5	455	1–5
Homosexuals working with children	1.45	0.05	1–5	459	1–5
Homosexual bars should be closed	1.25	0.03	1–3	458	1–5
Homosexuals and social equality	1.32	0.03	1–5	458	1–5
Homosexuals and equal opportunity employment	1.20	0.02	1–3	459	1–5
No reason to restrict where homosexuals work	1.29	0.03	1–5	460	1–5
Bar homosexuals from teaching profession	1.24	0.03	1–5	457	1–5
Homosexuals should be allowed to marry	1.61	0.06	1–5	459	1–5
Admission to medical school					
Highly qualified homosexual applicant	0.01	0.00	0–1	455	0–1
Highly qualified transgender applicant	0.02	0.01	0–1	456	0–1
Highly qualified HIV-positive applicant	0.05	0.01	0–1	457	0–1
Referral to a homosexual colleague who is a					
Pediatrician	0.03	0.01	0–1	458	0–1
General Surgeon	0.00	0.00	0–1	455	0–1
Psychiatrist	0.02	0.01	0–1	456	0–1
Radiation Oncologist	0.00	0.00	0–1	452	0–1
Referral to a transgender colleague who is a					
Pediatrician	0.05	0.01	0–1	455	0–1
General Surgeon	0.02	0.01	0–1	453	0–1
Psychiatrist	0.05	0.01	0–1	452	0–1
Radiation Oncologist	0.02	0.01	0–1	451	0–1
Referral to an HIV-positive colleague who is a					
Pediatrician	0.10	0.01	0–1	455	0–1
General Surgeon	0.25	0.02	0–1	450	0–1
Psychiatrist	0.04	0.01	0–1	456	0–1
Radiation Oncologist	0.04	0.01	0–1	454	0–1
Affective orientation (“feelings about”)					
Homosexual patients	0.07	0.02	0–1	458	0–1
Transgender patients	0.22	0.02	0–1	454	0–1
HIV-positive patients	0.13	0.02	0–1	458	0–1
Summary scales^b,c^					
HATH-8	1.38	0.03	1–3.5	460	1–5
HATH-8 POMP	9.66	0.77	0–62.5	446	0–100
MHS	0.14	0.04	0–6	448	0–6
MHS POMP	2.35	0.63	0–100	448	0–100
MTS	0.37	0.05	0–6	444	0–6
MTS POMP	6.23	0.88	0–100	444	0–100
HIVS	0.61	0.06	0–6	447	0–6
HIVS POMP	10.16	1.01	0–100	447	0–100

^a^ Highest values indicate most unfavorable attitude. Lowest values indicate most favorable attitude.

^b^ Intrinsic consistency reliability of scales (Cronbach's alpha): HATH-8 (0.86), MHS (0.57), MTS (0.69), and HIVS (0.75).

^c^ Spearman's rank correlation between HATH-8 POMP and the three other composite measures was 0.28 (MHS), 0.33 (MTS), and 0.32 (HIVS). Correlation of HIVS with MTS was 0.34 and with MHS was 0.32. Spearman's rho between MTS and MHS was 0.53.

HATH, Heterosexual Attitudes Toward Homosexuality; HIVS, HIV Stigma; MHS, Medical Homosexual Stigma; MTS, Medical Transgender Stigma; POMP, percent of maximum possible; SE, standard error of mean.

### Bivariate associations

Table 3 presents the bivariate associations among the attitudinal outcome scales (POMP transformed) and selected covariates. It should first be noted that mean POMP scores did not exceed 25% of maximum possible (most unfavorable) for any attitudinal outcome or any covariate comparison. Female respondents endorsed significantly less stigma than male respondents on three of the four outcome scales (HATH-8, MHS, and HIVS). Earlier graduates endorsed significantly more stigma than more recent graduates on HATH-8 and HIVS. Outcome means did not differ by practice setting, specialty, or SDCMS membership, although HIVS POMP was somewhat higher among SDCMS members than nonmembers (11.2 vs. 6.6, *p* = 0.052). Not unexpectedly, those who identified as homosexual or bisexual, or who declined to state their sexual orientation, endorsed less stigma on all composite outcome measures.

**Table 3.** Bivariate Associations of Attitude Scales with Selected Covariates

*Covariate*	*Scale*	*Covariate level*	*Mean*	*SE*	*95% CI*	*Test statistic*	p
Gender						*F*[2, 417]	
	HATH-8 POMP	Male	11.3	1.6	8.1–14.6	7.19	0.0009
		Female	4.6	0.9	2.8–6.4		
		Undisclosed	18.3	9.1	0.4–36.2		
	MHS POMP	Male	3.5	1.2	1.1–5.8	3.36	0.0357
		Female	0.3	0.2	0.0–0.7		
		Undisclosed	1.3	3.4	−5.4 to 8.0		
	MTS POMP	Male	7.7	1.6	4.6–10.9	1.39	0.2499
		Female	4.0	1.3	1.5–6.5		
		Undisclosed	3.6	8.8	−13.7 to 20.8		
	HIVS POMP	Male	12.0	1.6	8.9–15.2	3.37	0.0354
		Female	6.8	1.2	4.4–9.1		
		Undisclosed	7.4	17.3	−26.6 to 41.3		
Year of medical school graduation						*F*[4, 415]	
	HATH-8 POMP	1942–1982	11.5	1.8	8.1–15.0	4.53	0.0014
		1983–1994	10.1	1.5	7.2–13.1		
		1995–2005	6.8	1.5	3.8–9.8		
		2006–2021	4.3	1.4	1.5–7.0		
		Undisclosed	23.3	7.9	7.7–38.8		
	MHS POMP	1942–1982	2.4	0.7	1.1–3.7	1.97	0.0978
		1983–1994	1.7	0.7	0.3–3.0		
		1995–2005	2.7	2.0	−1.1 to 6.6		
		2006–2021	0.6	0.4	−0.2 to 1.3		
		Undisclosed	7.7	7.3	−6.6 to 22.0		
	MTS POMP	1942–1982	7.7	2.0	3.7–11.7	0.71	0.5828
		1983–1994	6.1	1.3	3.6–8.6		
		1995–2005	4.3	2.0	0.3–8.3		
		2006–2021	4.8	1.6	1.6–7.9		
		Undisclosed	13.9	8.9	−3.5 to 31.3		
	HIVS POMP	1942–1982	20.1	2.7	14.9–25.4	11.78	0.0000
		1983–1994	9.0	1.5	6.0–11.9		
		1995–2005	8.8	2.5	3.8–13.7		
		2006–2021	6.1	2.4	1.4–10.8		
		Undisclosed	1.0	1.4	−1.7 to 3.8		
Practice setting						*F*[3, 416]	
	HATH-8 POMP	Academic medicine	6.2	2.4	1.5–10.9	1.07	0.3626
		Integrated medical group	10.3	2.9	4.6–16.0		
		Other	8.5	1.6	5.5–11.6		
		Private practice	11.7	1.9	8.0–15.3		
	MHS POMP	Academic medicine	2.4	2.3	−2.1 to 6.8	0.22	0.8808
		Integrated medical group	2.6	1.4	−0.2 to 5.4		
		Other	1.7	0.6	0.6–2.8		
		Private practice	2.7	1.4	−0.1 to 5.5		
	MTS POMP	Academic medicine	5.9	3.3	−0.5 to 12.4	0.3	0.8229
		Integrated medical group	6.2	2.0	2.3–10.1		
		Other	7.9	2.0	3.9–11.9		
		Private practice	5.4	1.6	2.3–8.5		
	HIVS POMP	Academic medicine	6.5	1.8	3.0–10.0	2.39	0.0681
		Integrated medical group	6.6	2.5	1.7–11.5		
		Other	12.7	2.2	8.3–17.2		
		Private practice	11.2	2.2	6.8–15.5		
SDCMS membership						*F*[1, 418]	
	HATH-8 POMP	No	7.1	1.7	3.7–10.5	1.6	0.2066
		Yes	10.0	1.2	7.7–12.4		
	MHS POMP	No	1.0	0.5	0.1–1.9	2.3	0.1304
		Yes	2.8	1.1	0.7–4.8		
	MTS POMP	No	6.2	1.9	2.4–10.0	0.01	0.9432
		Yes	6.4	1.4	3.7–9.1		
	HIVS POMP	No	6.6	1.9	2.9–10.4	3.81	0.0516
		Yes	11.2	1.4	8.5–13.9		
Specialty						*F*[3, 416]	
	HATH-8 POMP	Primary care^a^	8.8	1.7	5.5–12.0	0.45	0.7159
		Surgical^b^	10.7	2.0	6.7–14.6		
		Psychiatry	6.1	3.4	−0.6 to 12.9		
		Other	9.4	3.0	3.5–15.2		
	MHS POMP	Primary care	2.8	1.3	0.3–5.3	0.51	0.6774
		Surgical	1.4	0.5	0.4–2.3		
		Psychiatry	1.2	1.2	−1.3 to 3.6		
		Other	3.0	2.4	−1.7 to 7.7		
	MTS POMP	Primary care	6.9	1.6	3.9–10.0	0.63	0.5952
		Surgical	4.6	1.2	2.3–6.9		
		Psychiatry	6.0	2.5	1.2–10.8		
		Other	7.6	3.6	0.5–14.7		
	HIVS POMP	Primary care	8.3	1.6	5.2–11.4	2.09	0.1007
		Surgical	15.0	2.8	9.5–20.5		
		Psychiatry	8.2	2.9	2.4–14.0		
		Other	7.4	2.0	3.5–11.3		
Sexual orientation						*F*[1, 418]	
	HATH-8 POMP	Heterosexual	10.1	1.0	8.2–12.0	5.95	0.0151
		Homosexual/bisexual/decline	2.3	3.0	−3.6 to 8.2		
	MHS POMP	Heterosexual	2.6	0.9	0.8–4.3	8.38	0.0040
		Homosexual/bisexual/decline	0.0				
	MTS POMP	Heterosexual	6.8	1.2	4.6–9.1	6.89	0.0090
		Homosexual/bisexual/decline	1.5	2.1	−2.7 to 5.8		
	HIVS POMP	Heterosexual	10.7	1.2	8.4–13.0	3.22	0.0735
		Homosexual/bisexual/decline	4.4	3.4	−2.2 to 11.1		

^a^ Primary care includes Family Medicine, General Practice, Internal Medicine, and Pediatrics.

^b^ Surgical includes General Surgery, Surgical subspecialties, Obstetrics and Gynecology, and Anesthesiology.

CI, confidence interval.

### Multivariable analyses

Table 4 presents the results of gamma GLM of the four composite outcome measures on those covariates found in the bivariate analysis to be associated with the outcomes: gender, year of medical school graduation, and sexual orientation. The effects in Table 4 are exponentiated model coefficients and are interpreted as the ratio of estimated outcome means comparing the index covariate category to the reference category. For example, for HATH-8 POMP, the exponentiated coefficient of 0.45 for female gender represents the estimated ratio of the HATH-8 POMP mean for females to the mean for males. In general, where effects were found, women, more recent graduates, and self-identifying homosexual or bisexual respondents scored toward the more favorable ranges of the composite outcome measures.

**Table 4.** Generalized Linear Models of Selected Covariates on Composite Percent of Maximum Possible Outcomes

*Covariate*	*HATH-8,*^a^*exp(b)*^e^*(SE)*	*MHS,*^b^*exp(b) (SE)*	*MTS,*^c^*exp(b) (SE)*	*HIVS,*^d^*exp(b) (SE)*
Gender				
Male	1.00 (.)	1.00 (.)	1.00 (.)	1.00 (.)
Female	0.45^***^ (0.10)	0.03^***^ (0.02)	0.53^*^ (0.16)	1.08 (0.29)
Missing/undisclosed	0.93 (0.54)	0.35 (0.46)	1.30 (1.45)	0.88 (0.72)
Year of graduation				
1942–	1.00 (.)	1.00 (.)	1.00 (.)	1.00 (.)
1983–	0.95 (0.18)	2.12 (2.12)	0.91 (0.31)	0.40^***^ (0.10)
1995–	0.62^*^ (0.14)	0.45 (0.34)	0.52 (0.26)	0.39^**^ (0.12)
2006–	0.47^*^ (0.15)	0.08^***^ (0.06)	0.85 (0.35)	0.34^**^ (0.13)
Missing	1.94^*^ (0.54)	1.25 (0.83)	2.14 (0.98)	0.04^**^ (0.05)
Sexual orientation		Not estimable		
Heterosexual	1.00 (.)	—	1.00 (.)	1.00 (.)
Homosexual/bisexual/decline	0.35^*^ (0.18)	—	0.18^**^ (0.12)	0.33^**^ (0.14)
Observations	446	448	444	447

(.) indicates that no confidence interval is presented because the indicated category is the reference category.

Because of marked skewness of composite outcomes, models were fit with gamma family distribution and log link.

^a^ HATH-8 POMP covariate *F* tests: (1) gender *F* (2, 444) = 7.00, *p* = 0.0010; (2) graduation year *F* (4, 442) = 4.76, *p* = 0.0009; (3) sexual orientation *F* (1, 445) = 4.25, *p* = 0.0398.

^b^ MHS POMP covariate *F* tests: (1) gender *F* (2, 446) = 10.26, *p* < 0.00001; (2) graduation year *F* (4, 444) = 3.64, *p* = 0.0062. Because all respondents who endorsed being homosexual or bisexual (or declined to state) scored 0 on the MHS POMP outcome, the full generalized linear model failed to converge. Therefore, a reduced model, omitting sexual orientation, was fit for MHS POMP.

^c^ MTS POMP covariate *F* tests: (1) gender *F* (2, 442) = 1.14, *p* = 0.3197; (2) graduation year *F* (4, 440) = 0.75, *p* = 0.5591; (3) sexual orientation *F* (1, 443) = 3.08, *p* = 0.0799.

^d^ HIVS POMP covariate *F* tests: (1) gender *F* (2, 445) = 0.05, *p* = 0.9494; (2) graduation year *F* (4, 443) = 5.91, *p* = 0.0001; (3) sexual orientation *F* (1, 446) = 6.79, *p* = 0.0095.

^e^ Exponentiated coefficients [exp(b)] are interpreted as the ratio of the arithmetic means, comparing the index covariate level with the reference level.

^*^ *p* < 0.05, ^**^p < 0.01, ^***^p < 0.001.

### Medically related attitudinal questions

Regarding associations between opposition to entry to medical school for homosexual, transgender, or HIV-positive individuals, relative to opposition to a homosexual applicant, a transgender applicant was 4.1 times more likely to be opposed (McNemar *p* = 0.044) and an HIV-positive applicant was 8.5 times as likely to be opposed (*p* < 0.0001). Considering feelings about treating patients, those endorsing being sometimes or often uncomfortable about treating homosexual patients were 3.1 times more likely to have similar feelings about treating transgender patients (*p* < 0.0001) and 1.8 times more likely to have those feelings about treating HIV-positive patients (*p* < 0.004). Finally, regarding referral to a pediatrician, those expressing intent to discontinue referral to a homosexual pediatrician were 1.7 times more likely to discontinue referral to a transgender pediatrician (*p* = 0.004) and 3.5 times as likely to discontinue referral to an HIV-positive pediatrician (*p* < 0.0001).

### Comparison with prior two survey waves

**FIG. 2.**
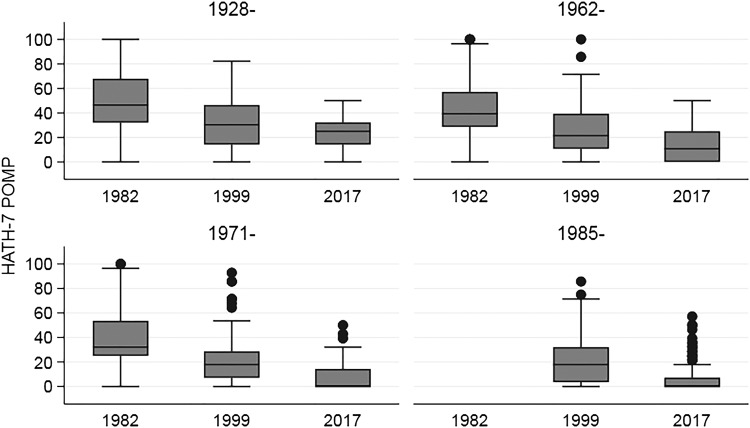
Distribution of unweighted HATH-7 POMP scores by survey wave (1982, 1999, and 2017), stratified by year of medical school graduation quartile. Test for equality of medians, by year of graduation quartile: 1928– (*p* < 0.0001), 1962– (*p* < 0.0001), 1971– (*p* < 0.0001), 1985– (*p* < 0.0001). POMP, percent of maximum possible score.

For the unweighted comparison of HATH-7 POMP scores across all three waves of the SDCMS (in 1982, 1999, and 2017), the median (interquartile range [IQR]) scores declined from 42.9 (28.6–64.3) in 1982 to 21.4 (7.1–32.1) in 1999 and to 0.0 (0.0–14.3) in 2017 (*p*_trend_ < 0.0001). Figure 2 presents box plots of HATH-7 POMP scores for the three survey waves, stratified by quartile of year of medical school graduation. The figure demonstrates that, although earlier graduates had higher scores (more stigma) than more recent graduates, within the same year of graduation quartile, respondent homosexual stigma decreased from 1982 to 2017. Opposition to entry of a highly qualified homosexual applicant to medical school declined from 30% in 1982 to 3% in 1999 and to 0.4% in 2017. Opposition to medical school entry of a highly qualified asymptomatic HIV-positive applicant with excellent response to antiretroviral therapy declined from 37% in 1999 to 6% in 2017.

Regarding consultative referral to specific specialties based on consultant characteristics, intended discontinuation of referral to homosexual pediatricians declined from 46% in 1982 to 9% in 1999 and to 2% in 2017. The prevalence of intended referral discontinuation to HIV-positive general surgeons declined from 59% in 1999 to 27% in 2017. Regarding predictors of HATH-7 POMP scores across the three survey waves, Table 5 presents the unweighted median (IQR) scores and Kruskal–Wallis *p* values by sexual orientation, gender, and year of graduation from medical school. Concordant associations were noted for all three covariates across the survey waves except for gender in wave 1 (1982). More recent graduates, self-identified homosexual or bisexual respondents, and women endorsed responses associated with less stigma.

**Table 5.** HATH-7 POMP Scores by Survey Year and Covariate Levels

	*Survey year*		
	*1982*	*1999*	*2017*
*Covariate*	*Median (IQR)*	*Median (IQR)*	*Median (IQR)*
Sexual orientation			
Heterosexual	—	21.4 (7.1–32.1)	0 (0–14.3)
Homosexual/bisexual/decline	—	17.9 (0–32.1)	0 (0–0)
KW *p* value	—	0.008	0.0002
Gender			
Male	42.9 (28.6–64.3)	21.4 (7.1–32.1)	3.6 (0–17.9)
Female	35.7 (25–66.1)	14.3 (3.6–28.6)	0 (0–5.4)
Missing	42.9 (35.7–71.4)	35.7 (7.1–50)	0 (0–21.4)
KW *p* value	0.27	0.006	0.0001
Year of graduation			
1928–	46.4 (32.1–67.9)	30.4 (14.3–46.4)	25 (14.3–32.1)
1962–	39.3 (28.6–57.1)	21.4 (10.7–39.3)	10.7 (0–25)
1971–	32.1 (25–53.6)	17.9 (7.1–28.6)	0 (0–14.3)
1985–	—	17.9 (3.6–32.1)	0 (0–7.1)
KW *p* value	0.0001	0.0001	0.0001

IQR, interquartile range; KW, Kruskal–Wallis.

## Discussion

Since the 1982 and 1999 PATHH surveys, there have been dramatic declines in the prevalence of self-reported stigmatizing attitudes toward sexual minorities and HIV-positive persons in the respondents to the SDCMS survey.^14,15^ Consistent predictors of greater sexual orientation stigma across the three survey waves were earlier year of medical school graduation, heterosexual orientation, and male gender. These associations should be understood in the context of significant declines in stigma among respondents with these same characteristics across the three study waves. While the PATHH-I survey noted that certain specialties, such as surgeons, endorsed greater stigma than other specialties,^14^ no difference by specialty was observed in the PATHH-III study. Attitudes toward transgender people were assessed only in the current survey. Although transgender stigma scores were low, they were higher than corresponding gay and lesbian (homosexual) stigma scores but lower than corresponding HIVS scores (Table 2). Matched analysis of association between responses dealing with entry to medical school, referral to pediatricians, and feelings about treating patients revealed that, relative to a gay or lesbian referent, endorsed stigma was amplified 1.7- to 4.1-fold for a transgender referent and 1.8- to 8.5-fold for an HIV-positive referent.

Overall, the results of the current survey, considering prior estimates from 1982 and 1999, mirror broad favorable shifts in Western societal attitudes toward sexual minorities and HIV-positive persons.^21–23^ We are unable to determine whether the more favorable attitudes observed in our most recent survey waves, in comparison to the 1982 survey, are attributable solely to general societal changes or also to concurrent enhancements in medical curricula and postgraduate training.^24^ While there are, no doubt, important differences in regional, cultural, and religious attitudes that limit generalizability of the findings, the current results provide some reassurance to physicians and patients who are themselves members of sexual minorities or HIV positive that stigma associated with these characteristics has declined markedly in a major urban medical community in California.

Surprisingly, there have been few similar surveys of physician attitudes toward sexual minorities as patients and colleagues. A New Mexico survey conducted in 1996 used questions from the 1982 San Diego study, allowing for direct comparison.^25^ The acceptability of a gay or lesbian individual being admitted to medical school and the intention to discontinue referral to a gay or lesbian pediatrician were closest to the estimates observed in the San Diego PATHH-II (1999) study (95.7% vs. 96.8% and 11% vs. 10%, respectively). The concordance of survey estimates in the two studies differing in time only by 3 years highlights the potential importance of both regional and temporal factors as determinants of the prevalence and degree of endorsed stigma in similar studies.

To what extent does endorsed stigma in surveys such as the current one predict either internalized self-stigma or expressed stigma by those who endorse stigma-associated responses? This question has been inadequately studied. However, the effect of sexual orientation and/or gender identity stigma is reflected in the reported experiences of LGBT practitioners in the literature. In one national survey, 8% of LGBT physicians reported that they were denied employment, 2% denied medical school entry, and 10% denied patient referral because of their sexual or gender identity.^26^ Perceived sexual and gender minority (SGM) inclusivity has also been shown to have an impact on choice of specialty. SGM physicians have tended to choose specialties that have more SGM persons in them and are perceived to be more inclusive, such as psychiatry, family medicine, pediatrics, and internal medicine.^27^ However, results from the current survey, when compared with the 1982 San Diego results, suggest that differences in specialty-associated sexual orientation stigma may be declining.

The strengths of the current research include the following: (1) it fills a gap in research regarding current attitudes of practicing physicians toward sexual and gender minorities and HIV-positive persons, especially regarding medical contexts; (2) it elucidates important trends and generational effects in attitudes of a major urban California medical community over a 35-year period; and (3) because of the availability of demographic characteristics of nonrespondents for the 2017 survey wave, nonresponse weighting could be applied to attenuate the effects of nonresponse bias. Future research should explore whether declines in stigma-associated attitudes among clinicians correspond to more favorable healthcare experiences for patients and are also associated with enhanced cultural and medical competencies. Moreover, it would be valuable to explore attitudinal heterogeneity among physicians differing by geographical region and other potentially salient stigma-related characteristics not assessed in this survey, such as religiosity and political affiliation.

### Limitations

The study has important limitations. First, the click-through response rate was only 11.6%. Although this is similar to the 13% response rate of the 1999 PATHH-II survey, it is substantively less than the 42.7% achieved in the 1982 PATHH-I survey and the 54.3% observed in the 1996 New Mexico survey.^25^ However, the 2017 PATHH-III survey was deployed as an Internet survey, whereas the earlier surveys were mail surveys. Mail surveys have, in some circumstances, been shown to have a four times higher response rate than those deployed online.^28^ The response rate difference has been attributed to potential concerns about confidentiality, ease of internet access, and databases of physician e-mail addresses not being as accurate or up to date as postal addresses.^29^

In addition, our response rate, defined as click-through rate, is somewhat better than the 6.93% rate reported for surveys of health professionals by Constant Contact as of March 2018.^30^ In addition, online surveys of the SDCMS membership conducted by the SDCMS between 2013 and 2016 had click-through rates varying between 1.8% and 9.9% (SDCMS, personal communication, November 7, 2017). With such low response rates, the risk for nonresponse bias is substantial. Nonetheless, by employing nonresponse weighting, we believe that the impact of nonresponse bias has been attenuated. Although we were not able to include sexual orientation and gender identity in nonresponse weights, it is reassuring that there were relatively similar proportions of self-reported LGBT respondents in the PATHH-III (8.9%) and PATHH-II (7%) surveys,^15^ although both proportions are somewhat higher than the 4.6% California LGBT population estimated by The Williams Institute.^31^

A second limitation is the generalizability of our findings. As a survey of a single urban California medical community, the results should not be generalized broadly and call for more representative state and national surveys of comparable content.

A third limitation is the knowledge gap that exists regarding the ability of endorsed stigma in surveys to predict both behavioral intention to discriminate and expressed or acted out stigma in patient care and professional relationships.

Fourth, we recognize that our use of the term “transgender” among respondent demographic questions (item 4) does not conform to the currently recommended procedures for ascertaining gender identity.^32^ Finally, our data do not directly address the relationship between stigma-associated attitudes and LGBT care competence, a multidimensional construct amenable to improvement through education, mentorship, and contact with LGBT persons.^33–35^

## Conclusions

We found substantive declines over a 35-year period in the prevalence of stigmatizing attitudes toward sexual minorities and HIV-positive people among physician respondents in three survey waves of the San Diego County medical community. More recent graduates, self-identified gay or bisexual respondents, and women endorsed responses associated with less stigma. But even among earlier graduates, analysis across all three survey waves showed impressive declines in endorsed stigma. Future research is needed to validate and extend our findings in more varied and representative physician samples.

## Appendix 1: PATHH-III Survey

### Introduction

You are being invited to participate in an anonymous research study entitled Physician's Attitudes Toward LGBT Individuals—PATHH-III Survey. This study is being conducted by Drs. Robert Marlin, Ankita Kadakia, and Christopher Mathews from the University of California, San Diego (UCSD). You were selected to participate in this study because of your membership in California's San Diego County Medical Society and/or because you are UCSD clinical faculty.

The purpose of this research study is to assess provider attitudes toward LGBT issues. We will also assess how these attitudes have changed over time using past surveys. If you agree to take part in this study, you will be asked to complete an online survey. This survey will ask about basic demographic information and your opinions on LGBT issues and will take approximately 5 minutes to complete.

There is no direct benefit to you from this research. The investigators, however, may learn more about attitudes toward LGBT issues in healthcare.

There are some risks associated with survey research including potential violation of confidentiality. Keeping the survey anonymous and collecting only minimal, nonidentifiable demographic data will minimize this risk. We encourage you to take the survey in a private setting to further safeguard anonymity. Research records will be kept confidential to the extent allowed by law and may be reviewed by the UCSD Institutional Review Board.

Your participation in this study is completely voluntary, and you can withdraw at any time by simply exiting the survey. Choosing not to participate or withdrawing will result in no penalty or loss of benefits to which you are entitled.

If you have questions about this project or if you have a research-related problem, you may contact the researcher Robert Marlin at rmarlin@ucsd.edu. If you have any questions concerning your rights as a research subject, you may contact the UCSD Human Research Protections Program Office at 858-246-HRPP (858-246-4777).

By clicking “I agree” below you are indicating that you are at least 18 years old, have read this consent form, and agree to participate in this research study. Please print a copy of this page for your records.

**(1) Do you agree to participate in this survey?***
() I agree, continue to the survey
() I do not agree, exit the survey after completing demographic questions.
___________________________________________
**Demographics**
**(2) What is your medical specialty***
() Allergy and Immunology
() Anesthesiology
() Colon and Rectal Surgery
() Dermatology
() Emergency Medicine
() Family Medicine
() Internal Medicine
() Medical Genetics and Genomics
() Neurological Surgery
() Plastic Surgery
() Nuclear Medicine
() Obstetrics and Gynecology
() Ophthalmology
() Orthopedic Surgery
() Osteopathic Neuromusculoskeletal Medicine
() Pathology
() Pediatrics
() Physical Medicine and Rehabilitation
() Preventative Medicine
() Psychiatry
() Radiation Oncology
() Radiology
() Surgery
() Thoracic Surgery
() Urology
() Neurology
() General Practice
() Other
**(3) What is your gender?***
() Male
() Female
() Nonbinary/third gender
() Decline to state
**(4) Do you identify as transgender?***
() Yes
() No
() Decline to state
**(5) What is your sexual orientation?***
() Heterosexual
() Homosexual or bisexual
() Decline to state
**(6) Year of graduation from medical school:**
___________________________________________
**(7) What is your practice setting?***
() Private Practice
() Academic Medicine
() Retired
() Integrated Medical Group (e.g., Permanente)
() Other
**(8) Are you a member of California's San Diego County Medical Society?***
() Yes
() No
() Unsure
___________________________________________
**General Attitudes Toward LGBT Issues**
**(9) It would be beneficial to society to recognize homosexuality as normal.**
() Strongly agree () Agree () Neutral () Disagree () Strongly disagree
**(10) Homosexuals should not be allowed to work with children.**
() Strongly agree () Agree () Neutral () Disagree () Strongly disagree
**(11) All homosexual bars should be closed down.**
() Strongly agree () Agree () Neutral () Disagree () Strongly disagree
**(12) Homosexuals should be given social equality.**
() Strongly agree () Agree () Neutral () Disagree () Strongly disagree
**(13) Homosexuals should have equal opportunity employment.**
() Strongly agree () Agree () Neutral () Disagree () Strongly disagree
**(14) There is no reason to restrict places where homosexuals work.**
() Strongly agree () Agree () Neutral () Disagree () Strongly disagree
**(15) Homosexuals should be barred from the teaching profession.**
() Strongly agree () Agree () Neutral () Disagree () Strongly disagree
**(16) Homosexuals should be allowed to marry.**
() Strongly agree () Agree () Neutral () Disagree () Strongly disagree
_________________________________________
**Medically Oriented LGBT Questions**
**(17) Should a highly qualified homosexual applicant be admitted to medical school?**
() Yes
() No
**(18) Should a highly qualified transgender applicant be admitted to medical school?**
() Yes
() No
**(19) Should a highly qualified HIV-positive but asymptomatic applicant, with excellent response to antiretroviral therapy, be admitted to medical school?**
() Yes
() No
**(20) Suppose you learned that a physician colleague is a homosexual. Would you continue to refer your patients to this physician if he or she worked in any of the following specialties:**
  **Table:**
  	*Yes, would continue to refer*	*No, would discontinue referral*
  Pediatrics	()	()
  General Surgery	()	()
  Psychiatry	()	()
  Radiation Therapy	()	()
**(21) Suppose you learned that a physician colleague is a transgender individual. Would you continue to refer your patients to this physician if he or she worked in any of the following specialties:**
  **Table:**
  	*Yes, would continue to refer*	*No, would discontinue referral*
  Pediatrics	()	()
  General Surgery	()	()
  Psychiatry	()	()
  Radiation Therapy	()	()
**(22) Suppose you learned that a physician colleague is HIV infected. Would you continue to refer your patients to this physician if he or she worked in any of the following specialties:**
  **Table:**
  	*Yes, would continue to refer*	*No, would discontinue referral*
  Pediatrics	()	()
  General Surgery	()	()
  Psychiatry	()	()
  Radiation Therapy	()	()
**(23) How do you feel about treating homosexual patients?**
() No negative feelings
() Sometimes uncomfortable
() Often uncomfortable
**(24) How do you feel about treating transgender patients?**
() No negative feelings
() Sometimes uncomfortable
() Often uncomfortable
**(25) How do you feel about treating HIV-positive patients?**
() No negative feelings
() Sometimes uncomfortable
() Often uncomfortable
___________________________________________
**Thank You!**
Thank you for taking our survey. Your response is very important to us.
___________________________________________
